# Enhanced Neural Cell Adhesion and Neurite Outgrowth on Graphene-Based Biomimetic Substrates

**DOI:** 10.1155/2014/212149

**Published:** 2014-01-30

**Authors:** Suck Won Hong, Jong Ho Lee, Seok Hee Kang, Eun Young Hwang, Yu-Shik Hwang, Mi Hee Lee, Dong-Wook Han, Jong-Chul Park

**Affiliations:** ^1^Department of Cogno-Mechatronics Engineering, Pusan National University, Busan 609-735, Republic of Korea; ^2^Education Program for Samsung Advanced Integrated Circuit, Pusan National University, Busan 609-735, Republic of Korea; ^3^Department of Maxillofacial Biomedical Engineering, School of Dentistry and Institute of Oral Biology, Kyung Hee University, Seoul 130-701, Republic of Korea; ^4^Cellbiocontrol Laboratory, Department of Medical Engineering, Yonsei University College of Medicine, Seoul 120-752, Republic of Korea

## Abstract

Neural cell adhesion and neurite outgrowth were examined on graphene-based biomimetic substrates. The biocompatibility of carbon nanomaterials such as graphene and carbon nanotubes (CNTs), that is, single-walled and multiwalled CNTs, against pheochromocytoma-derived PC-12 neural cells was also evaluated by quantifying metabolic activity (with WST-8 assay), intracellular oxidative stress (with ROS assay), and membrane integrity (with LDH assay). Graphene films were grown by using chemical vapor deposition and were then coated onto glass coverslips by using the scooping method. Graphene sheets were patterned on SiO_2_/Si substrates by using photolithography and were then covered with serum for a neural cell culture. Both types of CNTs induced significant dose-dependent decreases in the viability of PC-12 cells, whereas graphene exerted adverse effects on the neural cells just at over 62.5 ppm. This result implies that graphene and CNTs, even though they were the same carbon-based nanomaterials, show differential influences on neural cells. Furthermore, graphene-coated or graphene-patterned substrates were shown to substantially enhance the adhesion and neurite outgrowth of PC-12 cells. These results suggest that graphene-based substrates as biomimetic cues have good biocompatibility as well as a unique surface property that can enhance the neural cells, which would open up enormous opportunities in neural regeneration and nanomedicine.

## 1. Introduction

Graphene is a single-atom thick and is defined as a two-dimensional sheet of hexagonally arranged carbon atoms isolated from its three-dimensional parent material, graphite [1]. As with many novel materials, applications of graphene and its family nanomaterials, such as graphene oxide (GO), reduced GO (rGO), and graphene nanosheets, offer many technological opportunities since they exhibit interesting electrical, thermal, mechanical, and optical properties [2]. The practical uses of graphene family nanomaterials are extensive, covering applications as diverse as battery electrodes, super-capacitors, nanoelectronics (e.g., transistors and sensors), antibacterial paper, and many biomedical uses for drug delivery, diagnosis, and therapy [3–7]. These numerous potential applications of graphene and related materials make them very attractive to both the scientific and industrial community. However, to ensure the safe development of graphene and its family nanomaterials, their potential impact on health and environment remains unelucidated yet.

Carbon nanotubes (CNTs) and graphene, despite both being carbon-based, are two very distinct nanomaterials, and their biological applications still keep wide open. During the last decade, many studies of interactions between neural cells and carbon nanomaterials (CNMs) including CNTs, graphene, and their derivatives were carried out with terminally differentiated primary cells or cell lines [8, 9]. The primary focuses of very recent studies were on establishing biocompatibility and biofunctionality of the proposed materials, revealing that by pretreating rats with amine-modified single-walled CNTs (SWCNTs) neurons could be protected and the recovery of behavioural functions in rats with induced stroke could be enhanced [10], and graphene substrates exhibited excellent biocompatibility and significantly promoted neurite sprouting and outgrowth of mouse hippocampal cells [11].

In the present study, the biocompatibility between neural cells and three CNMs, namely, graphene, SWCNTs, and multiwalled CNTs (MWCNTs), was evaluated and compared by quantifying metabolic activity, intracellular oxidative stress, and membrane integrity. Neural cell adhesion and neurite outgrowth were examined onto graphene-based biomimetic substrates.

## 2. Experimental

### 2.1. Synthesis and Morphological Observation of Carbon Nanomaterials (CNMs)

Graphene and SWCNTs were grown by using chemical vapor deposition (CVD), as previously described [12, 13]. MWCNTs were synthesized by using spray pyrolysis combined with a subsequent thermal CVD process, as described elsewhere [14, 15]. After being synthesized, each CNM was weighed by using an electronic balance (with a readability of 0.1 mg, Adventurer Analytical Balance, Ohaus, Bradford, MA). The surface morphology of each CNM was observed by using scanning electron microscopy (SEM). In brief, all CMNs were coated with an ultrathin layer of gold/platinum by an ion sputter (E1010, Hitachi, Tokyo, Japan) and were then observed with a field emission scanning electron microscope (FESEM, Hitachi S-4700) at an accelerating voltage of 5 kV for graphene and 15 kV for both CNTs. A colloidal dispersive solution of each CNM was prepared in Dulbecco's phosphate-buffered saline (DPBS, Sigma-Aldrich Co., St Louis, MO, pH 7.4) with a final concentration of 500 ppm and was then sonicated for homogenous dispersions under mild conditions by using a water bath sonicator with a bath temperature of 25°C overnight. For biocompatibility evaluations, the suspension of each CNM was serially diluted with 2 × Dulbecco's modified Eagle's medium (DMEM, Sigma-Aldrich Co.) and was then treated to the cultured monolayer of neural cells.

### 2.2. Preparation of Graphene-Based Substrates

Graphene films were grown on catalytic copper (Cu) surface by using a CVD method [12, 13]. For the preparation of a graphene-coated substrate, the grown graphene film on a Cu foil was transferred onto a glass coverslip by using the scooping process. In detail, 10 wt% of poly(methyl methacrylate) (PMMA, Sigma-Aldrich Co.) was spin-casted on a Cu foil at 3000 rpm for 30 seconds and was then placed into an Cu etchant solution (Transene Company, Inc., Danvers, MA) to completely remove the Cu foil. Next, graphene covered with a PMMA substrate was scooped onto a glass coverslip, followed by removal of the PMMA by adding acetone for 40 min. For the neural cell adhesion, the top surface of graphene coated on the glass coverslip was covered with 50% fetal bovine serum (FBS, Sigma-Aldrich Co.) for 1 hour while shaking at 37°C and 80 rpm [16]. In the case of the graphene-patterned substrate, a patterned array of graphene films with rectangular shapes (100 *μ*m × 150 *μ*m) was fabricated on a SiO_2_/Si substrate by using a conventional photolithography (AZ 5214) as described elsewhere [12]. The morphology of the patterned graphene array was observed under a scanning electron microscope (SEM, Hitachi S-4800, Tokyo, Japan) at an accelerating voltage of 1.0 kV. To observe the neurite outgrowth, the top surface of the patterned graphene was covered with FBS by using the same method as mentioned above.

### 2.3. Cell Cultures and Conditions

PC-12 cells (derived from pheochromocytoma of rat adrenal medulla) were obtained from American Type Culture Collection (ATCC, Rockville, MD). Cells were routinely maintained in RPMI-1640 media (Sigma-Aldrich Co.) supplemented with 10% horse serum, 5% FBS, and 1% antibiotic antimycotic solution (including 10,000 U penicillin, 10 mg streptomycin, and 25 *μ*g amphotericin B per mL, Sigma-Aldrich Co.) at 37°C in a humidified atmosphere of 5% CO_2_ in air.

### 2.4. WST-8 Assay for Metabolic Activity Determination

The number of viable cells was quantified indirectly by using highly water-soluble tetrazolium salt (WST-8, 2-(2-methoxy-4-nitrophenyl)-3-(4-nitrophenyl)-5-(2,4-disulfophenyl)-2*H*-tetrazolium, monosodium salt; Dojindo Lab., Kumamoto, Japan), reduced to a water-soluble formazan dye by mitochondrial dehydrogenases. The cell viability was found to be directly proportional to the metabolic reaction products obtained in WST-8 [17]. Briefly, the WST-8 assay was conducted as follows: PC-12 cells were treated with increasing concentrations (0.5~500 ppm) of each CNM and were then incubated with WST-8 reagent for the last 4 hours of the culture period (24 hours) at 37°C in the dark. Parallel sets of wells containing freshly cultured cells, which were not treated with any CNMs suspended in the same concentration ratio of DPBS and DMEM, were regarded as negative controls. The absorbance was determined at 450 nm by using an ELISA reader (SpectraMax 340, Molecular Device Co., Sunnyvale, CA). The relative cell viability was determined as the percentage ratio of the optical densities in the media (containing CNMs at each concentration) to that of the fresh control medium.

### 2.5. DCF Assay for Oxidative Stress Determination

The 2′,7′-dichlorodihydrofluorescein (DCF) assay is a widely used method to detect intracellular reactive oxygen species (ROS) levels in pharmacological studies [18, 19]. The accumulation of intracellular free radicals from CNMs was quantified using a ROS assay kit (OxiSelect, Cell Biolabs, Inc., San Diego, CA), which employs the cell-permeable fluorogenic probe 2′,7′-dichlorodihydrofluorescein diacetate (DCFH-DA). DCFH-DA is an ROS detector that can cross cell membranes and be deacetylated by intracellular esterases to nonfluorescent 2′,7′-dichlorodihydrofluorescein (DCFH). In the presence of ROS, DCFH is rapidly oxidized to the highly fluorescent DCF, which is readily detectable. The fluorescence intensity is proportional to the ROS levels within the cell cytosol. PC-12 cells were exposed to increasing concentrations (0.5~500 ppm) of each CNM for 24 h and were then incubated with DCHF-DA for 30 minutes at 37°C in the dark. Parallel sets of wells containing freshly cultured cells, which were not treated with any CNMs suspended in the same concentration ratio of DPBS and DMEM, were regarded as negative controls. The fluorescence emission of DCF was monitored at regular intervals at an excitation wavelength of 480 nm and an emission wavelength of 530 nm in a fluorescence plate reader (VICTOR3 Multilabel Counter, PerkinElmer, Inc., Waltham, MA). The amount of DCF formed was calculated from a calibration curve constructed using an authentic DCF standard. The relative DCF intensity was determined as the percentage ratio of the fluorescence intensities in the wells (containing CNMs at each concentration) to that in the fresh control well.

### 2.6. LDH Assay for Membrane Integrity Determination

Cell membrane integrity was monitored using a lactate dehydrogenase (LDH) assay kit (Takara Bio Inc, Shiga, Japan) to determine the release of LDH into the medium according to the manufacturer instructions. In this assay, LDH released from damaged cells oxidizes lactate to pyruvate, which promotes conversion of the tetrazolium salt INT to a water-soluble red formazan product [19]. Briefly, after 24 hours exposure to increasing concentrations (0.5~500 ppm) of each CNM, the supernatant from each well was transferred to a new 96-well plate. Reconstituted substrate mix was added to each well and the plates were kept for 30 minutes in the dark at room temperature. Stop solution was then added to each well. Parallel sets of wells containing freshly cultured cells, which were not treated with any CNMs suspended in the same concentration ratio of DPBS and DMEM, were regarded as negative controls. Released LDH catalyzed the oxidation of lactate to pyruvate with simultaneous reduction of NAD^+^ to NADH. The rate of NAD^+^ reduction was directly proportional to LDH activity in the cell medium. The intensity of red color formed in the assay was measured at a wavelength of 490 nm with an ELISA reader (SpectraMax 340, Molecular Device Co.), which was proportional to the number of damaged cells. The relative LDH release was determined as the percentage ratio of the optical densities in the media (containing CNMs at each concentration) to that of the fresh control medium.

### 2.7. Assays for Neural Cell Adhesion, Neurite Outgrowth, and Proliferation

The adhesion of PC-12 cells and their neurite outgrowth were investigated onto graphene-coated and graphene-patterned substrates, respectively, under the conditions of the culture media without neural growth factors for neuronal differentiation. Neural cells were seeded with high density of 2 × 10^5^ cells/mL onto glass coverslips with FBS-covered graphene on them lying in a 48-well plate and were then incubated for 3 days. After incubation, cellular morphology adhered onto graphene-coated substrates was observed under an inverted microscope (IX81-F72, Olympus Optical, Osaka, Japan). For observing neurite outgrowth, PC-12 cells (low density of initial seeding, 1 × 10^4^ cells/mL) were cultured for 7 days onto FBS-covered graphene patterned on a SiO_2_/Si substrate lying in a 12-well plate. After cultivation, neurite outgrowth onto graphene-patterned substrate was visualized by using atomic force microscopy (AFM, Innova, Veeco Instruments Inc., Plainview, NY). In order to compare the proliferation pattern of PC-12 cells, cells were seeded on glass coverslips without and with FBS-covered graphene on them and then cultivated for 1, 3, 5, and 7 days at 37°C in a CO_2_ incubator. After incubation, the cell proliferation was determined by the WST-8 assay as described above.

### 2.8. Statistical Analysis

All variables were tested in three independent cultures for each cytotoxicity assay, which was repeated twice (*n* = 6). Quantitative data are expressed as mean ± standard deviation (SD). Data were tested for homogeneity of variances using Levene's test, prior to statistical analysis. Multiple comparisons to detect the dose-dependent effects of CNMs on PC-12 cells were carried out using one-way analysis of variance (ANOVA, SAS Institute, Cary, NC), which was followed by the Bonferroni test when variances were homogeneous and the Tamhane test when variances were not. Statistical analysis for the proliferation study was made by using the Student's *t*-test. A value of *P* < 0.05 was considered statistically significant.

## 3. Results and Discussion

### 3.1. SEM Analysis

**Figure 1:**
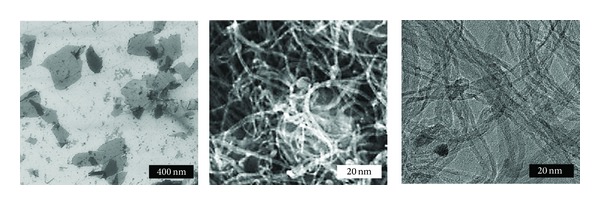
FESEM images of the surface morphologies of graphene nanoplatelets, SWCNTs, and MWCNTs.

Figure 1 shows FESEM images of pristine graphene, SWCNTs, and MWCNTs. All the CNMs were well dispersed in the culture medium (DMEM) with serum. Most of graphene nanoplatelets existed as single or few layers and presented both large and small sheets. Several graphene nanoplatelets with lateral sizes of around 200~500 nm have been observed while a few nanoplatelets showed smaller sizes within the range of 50~100 nm. SWCNTs and MWCNTs mostly formed nanofibrous bundles of 2~5 nm and 10~15 nm in diameter, respectively, and several *μ*m in length in the suspension.

### 3.2. Effects of CNMs on Metabolic Activity

**Figure 2:**
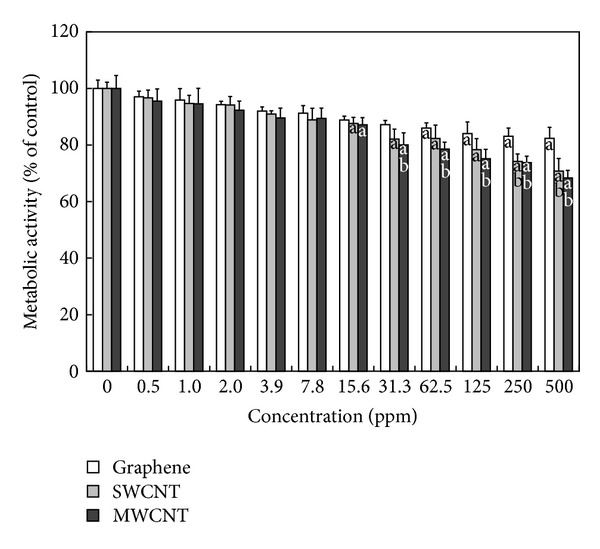
Effects of graphene, SWCNTs, and MWCNTs on mitochondrial toxicity of PC-12 cells. Cells were treated with different concentrations of CNMs for 24 hours. At the end of the incubation period, the WST-8 assay was performed to evaluate the cytotoxicity as described in Section 2. Data were expressed as mean ± standard deviation (SD) based on at least duplicate observations from three independent experiments. The letter “a” indicates statistically significant difference from the untreated control; the letter “b” indicates statistically significant difference from cells treated with graphene at the same concentration (*P* < 0.05).

In order to evaluate the neural cell biocompatibility of CNMs, the effects of CNMs on the metabolic activity of PC-12 cells were examined with the WST-8 assay where the formation of formazan dye depends on the mitochondrial enzyme activity. As shown in Figure 2, the viability of PC-12 cells decreased in a dose-dependent manner after 24 hours of exposure to increasing concentrations of each CNM. Graphene started to record significant (*P* < 0.05) mitochondrial toxicity from 62.5 ppm and showed about 18% loss in the cell viability even at the top concentration tested (500 ppm) in comparison to unexposed controls. In contrast, significant (*P* < 0.05) cytotoxicity was induced at 31.3 ppm of both CNTs, which resulted in approximately 18%~20% inhibition of the viability in comparison to untreated controls. A recent study reported that GO showed stronger hemolytic activity against red blood cells than aggregated graphene sheets whereas compacted graphene sheets were more damaging to mammalian fibroblasts than less densely packed GO [20]. Moreover, it was revealed that 7.5~30 ppm of SWCNTs reduced the total DNA content of mixed neuroglial cultures [21]. MWCNTs have been shown to induce massive loss of cell viability in human dermal fibroblasts through cell cycle arrest in the G_1_ phase, downregulation of adhesion-related genes, DNA damage, and programmed cell death as well as cause cytoskeleton damage and disturbance of actin stress fibers in the range of 40~200 ppm [22, 23]. In addition, our previous study revealed that primary-cultured fibroblasts were more susceptible to CNMs than the fibroblast cell line [24]. As a result, it is considered that the WST assay has any detection limit to find out cytotoxic effects of CNMs at relatively low concentrations (<31.3 ppm) on neural cells. Numerous previous studies have employed the methylthiazolyldiphenyl-tetrazolium bromide (MTT) assay, a typical nanotoxicity assay, but this assay sometimes failed to predict the toxicity of CNMs because of the spontaneous reduction of MTT by CNMs, resulting in a false positive signal [20, 25]. Therefore, cytotoxicity against cells exposed to CNMs should be also determined by alternative *in vitro* cell endpoint assays, such as ROS production, lipid peroxidation, and LDH leakage, because a WST-8 assay is based only on the activity of mitochondrial dehydrogenases.

### 3.3. Effects of CNMs on Intracellular Oxidative Stress

**Figure 3:**
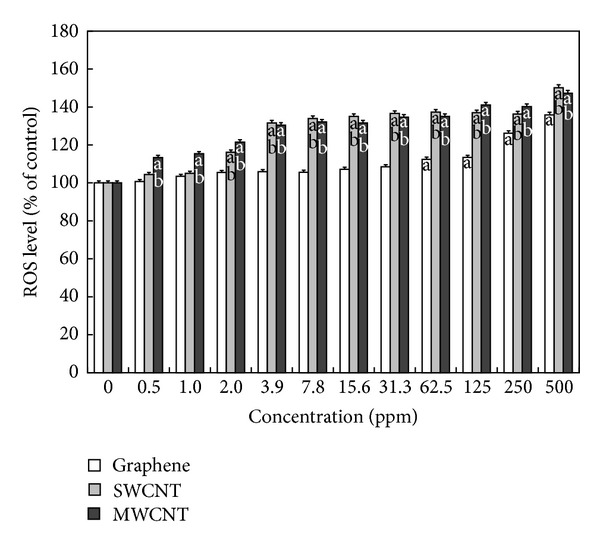
Effects of graphene, SWCNTs, and MWCNTs on ROS generation in PC-12 cells. Cells were treated with different concentrations of CNMs for 24 hours. At the end of the incubation period, the DCF assay was performed to evaluate the cytotoxicity as described in Section 2. Data were expressed as mean ± standard deviation (SD) based on at least duplicate observations from three independent experiments. The letter “a” indicates statistically significant difference from the untreated control; the letter “b” indicates statistically significant difference from cells treated with graphene at the same concentration (*P* < 0.05).

The DCF assay has been well verified as an effective index for evaluating the toxicity of nanomaterials attributable to ROS generation [19, 26]. Following exposure of PC-12 cells for 24 hours to each CNM, the state of oxidative stress in the cells was observed. As shown in Figure 3, the ROS generation increased in a dose-dependent manner as the concentration of each CNM increased, with the exception of graphene at the concentrations lower than 125 ppm. However, significant (*P* < 0.05) ROS generation started to be recorded from 3.9 ppm of both CNTs, which resulted in 130% increase in comparison to unexposed controls. These results roughly correlated with results from the WST-8 assay, suggesting that toxicity in cells exposed to CNTs might result from oxidative stress mediated by ROS generated from CNTs internalized into cells [9]. There is convincing evidence supporting this suggestion. It was demonstrated that long SWCNTs led to significant increases in ROS generation and malondialdehyde (a product of lipid peroxidation) level in PC-12 cells in time and dose-dependent manners [27]. Other evidence showed that exposure to MWCNTs resulted in a concentration-dependent cytotoxicity in cultured human embryonic kidney cells, which was associated with increased oxidative stress [28]. On the other hand, surface functionalization (e.g., PEGylation) of SWCNTs has been shown to decrease ROS-mediated toxicological response in PC-12 cells [29]. Furthermore, it was reported that vitamin E might protect PC-12 cells from the injury induced by SWCNTs through the downregulation of oxidative stress and prevention of mitochondrial-mediated apoptosis [30].

### 3.4. Effects of CNMs on Cell Membrane Integrity

**Figure 4:**
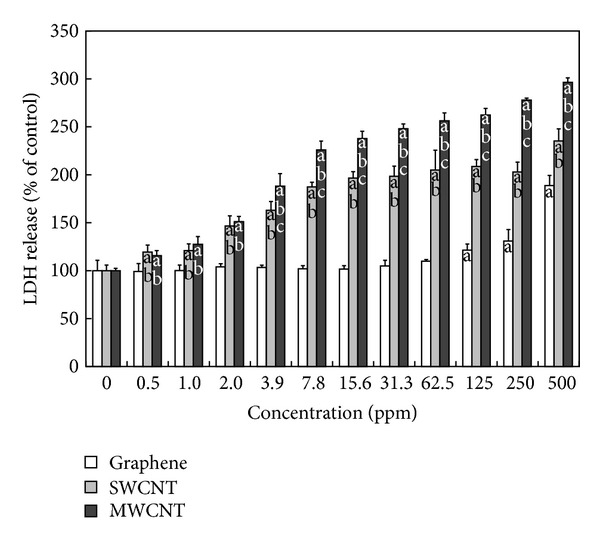
Effects of graphene, SWCNTs, and MWCNTs on LDH release from PC-12 cells. Cells were treated with different concentrations of CNMs for 24 hours. At the end of the incubation period, the LDH assay was performed to evaluate the cytotoxicity as described in Section 2. Data were expressed as mean ± standard deviation (SD) based on at least duplicate observations from three independent experiments. The letter “a” indicates statistically significant difference from the untreated control; the letters “b” and “c” indicate statistically significant differences from cells treated with graphene and SWCNTs, respectively, at the same concentration (*P* < 0.05).

LDH leakage is well known as a useful index for cytotoxicity on the basis of loss of membrane integrity, a hallmark of necrosis. All the CNMs induced apparent LDH release from PC-12 cells, revealing the adverse effect of CNMs on cell membrane integrity (Figure 4). Significant LDH release was noted only after 24 hours of exposure to graphene at higher concentrations (250 and 500 ppm). At lower concentrations (0.5~125 ppm), graphene had no effect on the release of LDH. In contrast, both SWCNTs and MWCNTs began to induce a significant (*P* < 0.05) increase in LDH release from 0.5 ppm and resulted in 235% and 296%, respectively at the highest concentration (500 ppm) in comparison to untreated controls. Some reasons can be evoked to explain the difference in cytotoxicity between graphene and CNTs. Generally, the size (namely, dimensions), shape, composition, surface charge, and surface chemistry (e.g., functionalization) of nanomaterials as well as the target cell type are critical determinants of intracellular responses, degree of cytotoxicity and potential mechanisms of toxicity [31]. The chemical composition and dimensions of graphene are similar to those of CNTs, but the shape of graphene is completely different from that of CNTs (planar versus cylindrical) [32]. Thus, it is likely that the piercing, needle-like CNT may be more mobile than the sheet-like graphene and can more readily penetrate the cell membrane, resulting in greater cell membrane damage [33]. These dose-dependent responses of PC-12 cells to CNMs correlated exactly with those from the DCF assay, implying that cell membrane damage is another mechanism for the toxicity of CNMs. In this study, the >250 ppm of graphene increased the LDH release and ROS generation. However, lower doses (0.5~31.3 ppm) of graphene had no effect on multiple endpoints such as metabolic activity, LDH leakage, and ROS production. Therefore, lower levels of exposure (<30 ppm) to graphene could theoretically be useful in biomedical applications including imaging, drug delivery, tissue engineering, and biosensors [34, 35]. Future studies will focus on the mechanistic studies regarding the interaction between CNMs and immune cells or tissues in order to ensure that these materials are developed in a safe and responsible manner to help confirm their long-term sustainability.

### 3.5. Neural Cell Adhesion and Neurite Outgrowth on Graphene-Based Biomimetic Substrates

**Figure 5:**
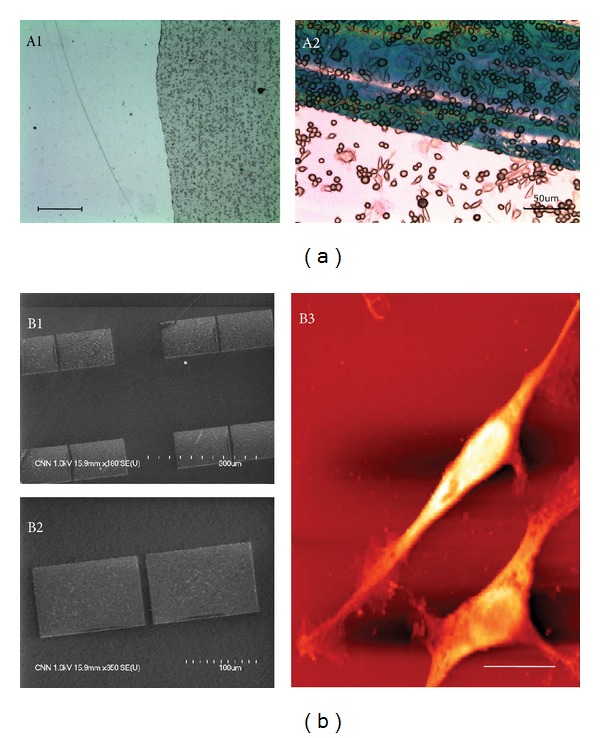
Neural cell adhesion (a) and neurite outgrowth (b) on graphene-based biomimetic substrates. Bright-field images of a glass coverslip with FBS-covered graphene on it (A1, scale bar = 10 *μ*m) and PC-12 cells on the boundary area between glass (lower) and graphene (upper) 3 days after cell culture (A2). SEM images (B1 and B2) of graphene patterned on a SiO_2_/Si substrate (B2, enlarged image of B1) and AFM image of neurite outgrowth from PC-12 cells on patterned graphene covered with FBS after 7 days of incubation (B3, scale bar = 20 *μ*m).

**Figure 6:**
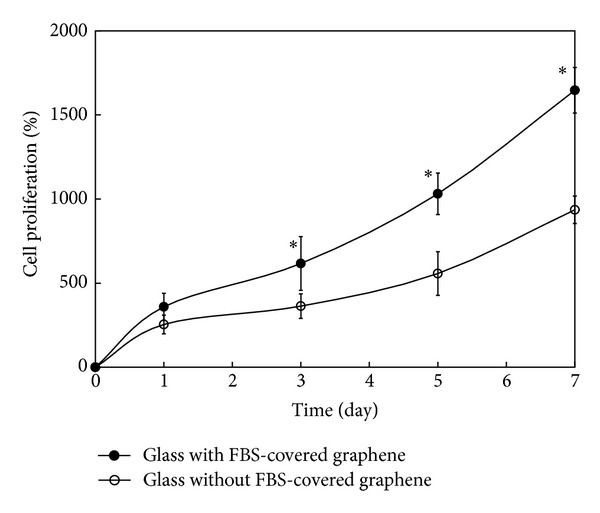
Proliferation of PC-12 cells cultured on bare glass coverslips with and without FBS-covered graphene on them after 1, 3, 5, and 7 days of incubation. At the end of each incubation period, the WST-8 assay was performed to examine the cell proliferation as described in materials and methods. Data were expressed as mean ± standard deviation (SD) based on at least duplicate observations from three independent experiments. The asterick denotes significant difference in the proliferation between bare glass coverslips with and without FBS-covered graphene, *P* < 0.05.

After PC-12 cells were cultured on a glass coverslip with FBS-covered graphene on it, their morphology was observed by using the optical microscopy. Cells were able to grow under the conditions of culture media without neural growth factors for neuronal differentiation. As shown in Figure 5(a), more cells were found to be adhered on the glass coverslip with FBS-covered graphene on it than on the glass coverslip without graphene-coated layers after 3 days of incubation. Moreover, PC-12 cells adhered on the bare glass coverslip appeared to partly take a spindle shape, while the graphene-coated substrate did not seem to have the same effect on cells. This pattern in the cellular adhesion was in good agreement with our previous study showing that adhesion and proliferation of PC-12 cells cultured onto graphene-coated glass coverslips were superior to those onto uncoated ones [16]. It has been reported that NIH-3T3 fibroblasts, although the cell type is different from neural cells, show highly improved cell growth, adhesion, and gene transfection efficiency on rGO/MWCNT-coated substrates [36]. Another report has revealed that rGO is biocompatible with PC-12 cells, whereas the SWCNT network is inhibitory to the proliferation, viability, and neuritegenesis of PC-12 cells [37]. This contrasting phenomenon was explained by the hypothesis that could be attributed to the distinct nanotopographic features of these two kinds of nanocarbon substrates. Controlling microenvironments of cells on certain substrates makes it possible to mimic *in vivo* situations and consequently contributes to the differentiation of stem cells into specific cell types [38]. On the other hand, PC-12 cells were shown to spread with apparent neurite outgrowth on the patterned graphene covered with FBS after 7 days of incubation (Figure 5(b)). As shown in Figure 6, PC-12 cells were cultured on glass coverslips without and with FBS-covered graphene on them, and their proliferation was examined by using the WST-8 assay. The time-dependant proliferation pattern of PC-12 cells on the glass coverslip with FBS-covered graphene on it was almost similar to that of the cells on the bare glass coverslip. However, PC-12 cells on the glass coverslip with FBS-covered graphene on it better proliferated than on the bare glass coverslip. The differentiation of PC-12 cells could be initiated simply by exchanging culture media without any neural growth factors for neuronal differentiation. This result suggests that graphene-patterned substrates as biomimetic cues have a specific surface property that can promote neural cells. Recent evidence supports this suggestion, showing that behaviors of neural stem cells, such as attachment, proliferation, and differentiation on the surface-functionalized graphene with laminin, were significantly better than those of the pure graphene surface [39]. In addition to this evidence, it has been reported that CNTs enhance the excitability of neurons by forming tight contacts with the cell membrane so that electrical activity is diverted through the nanotubes [40, 41].

## 4. Conclusion

From evaluation of the biocompatibility between neural cells and CNMs, it was demonstrated that graphene exerted much less adverse effects on neural cells than both types of CNTs, namely, SWCNTs and MWCNTs, at the concentrations lower than 62.5 ppm. Graphene-coated or graphene-patterned substrates were shown to substantially enhance the adhesion, neurite outgrowth, and proliferation of neural cells. Therefore, it is concluded that graphene-based biomimetic substrates have good biocompatibility as well as a unique surface property that can enhance neural cells, which would be potentially applied to neural regeneration and nanomedicine.
